# Predicting the Location and Spatial Extent of Submerged Coral Reef Habitat in the Great Barrier Reef World Heritage Area, Australia

**DOI:** 10.1371/journal.pone.0048203

**Published:** 2012-10-30

**Authors:** Tom Bridge, Robin Beaman, Terry Done, Jody Webster

**Affiliations:** 1 School of Earth and Environmental Sciences, James Cook University, Townsville, Queensland, Australia; 2 ARC Centre of Excellence for Coral Reef Studies, James Cook University, Townsville, Queensland, Australia; 3 School of Earth and Environmental Sciences, James Cook University, Cairns, Queensland, Australia; 4 Australian Institute of Marine Science, Townsville MC, Queensland, Australia; 5 Geocoastal Research Group, School of Earth Sciences, University of Sydney, Sydney, New South Wales, Australia; Heriot-Watt University, United Kingdom

## Abstract

**Aim:**

Coral reef communities occurring in deeper waters have received little research effort compared to their shallow-water counterparts, and even such basic information as their location and extent are currently unknown throughout most of the world. Using the Great Barrier Reef as a case study, habitat suitability modelling is used to predict the distribution of deep-water coral reef communities on the Great Barrier Reef, Australia. We test the effectiveness of a range of geophysical and environmental variables for predicting the location of deep-water coral reef communities on the Great Barrier Reef.

**Location:**

Great Barrier Reef, Australia.

**Methods:**

Maximum entropy modelling is used to identify the spatial extent of two broad communities of habitat-forming megabenthos phototrophs and heterotrophs. Models were generated using combinations of geophysical substrate properties derived from multibeam bathymetry and environmental data derived from Bio-ORACLE, combined with georeferenced occurrence records of mesophotic coral communities from autonomous underwater vehicle, remotely operated vehicle and SCUBA surveys. Model results are used to estimate the total amount of mesophotic coral reef habitat on the GBR.

**Results:**

Our models predict extensive but previously undocumented coral communities occurring both along the continental shelf-edge of the Great Barrier Reef and also on submerged reefs inside the lagoon. Habitat suitability for phototrophs is highest on submerged reefs along the outer-shelf and the deeper flanks of emergent reefs inside the GBR lagoon, while suitability for heterotrophs is highest in the deep waters along the shelf-edge. Models using only geophysical variables consistently outperformed models incorporating environmental data for both phototrophs and heterotrophs.

**Main Conclusion:**

Extensive submerged coral reef communities that are currently undocumented are likely to occur throughout the Great Barrier Reef. High-quality bathymetry data can be used to identify these reefs, which may play an important role in resilience of the GBR ecosystem to climate change.

## Introduction

Coral reefs, along with tropical rainforests, support greater biodiversity than any other ecosystem on earth. However, coral reefs worldwide are in decline from multiple threats including coastal development, over-fishing, land-based pollution and climate change [1]–[3]. Rising sea temperatures have resulted in mass bleaching and mortality of reef corals in recent decades [4], however deeper “mesophotic” reef habitats may be buffered from the synergistic effects of light and heat stress which cause corals to bleach [5], [6]. Therefore, deeper reef habitats (known as mesophotic coral reef ecosystems or MCEs) may therefore provide vital refugia for corals and associated species in coming decades [7], [8]. Unlike true deep-water coral reefs which occur in cold water and do not rely on sunlight for energy [9], mesophotic coral reefs occur in the middle to lower photic zone and often support rich communities of shallow-water corals and other photosynthetic taxa [10], [11]. However, MCEs have received little research effort compared to their shallow-water counterparts, largely due to their inaccessibility to traditional SCUBA surveys. Recent technological developments such as autonomous underwater vehicles (AUV) and remotely operated vehicles (ROV) have led to a substantial increase in MCE research in recent years [10]–[14] in recognition of both their unique biodiversity and their potential role as refugia. Despite their potential importance, basic information on the location and spatial extent of MCEs, particularly those occurring on submerged reefs too deep to be detected by airborne sensors, is not available in any of the world’s major coral reef regions.

Marine Protected Areas (MPAs) have become an important management tool for conserving coral reefs from climate change and other human impacts [15], [16]. However, the effectiveness of any protected area is contingent upon identifying 1) a representative sample of habitat types, and 2) areas that best protect species and ecosystems from processes that threaten their existence [17]. On coral reefs, biodiversity of both corals and reef fish often peaks in intermediate depths of 15–35 m [18]–[21], and deeper habitats are likely to be more stable and more likely to protect coral reef biota from threats such as rising sea temperatures and increased tropical storms [6], [8]. However, the vast majority of research on coral reefs is conducted in shallow water, often less than 10 m deep. Therefore, information used to inform the design of MPAs is heavily biased towards well-studied shallow habitats, and this may reduce the effectiveness of MPAs to perform these two functions.

Physical and environmental drivers of species distributions can be used as surrogates to predict the potential distribution of benthic marine ecosystems across large spatial scales and to identify priority sites for management [22], [23]. Although direct observations of MCEs are difficult and often expensive, predictive habitat models may provide a valuable tool to identify the location and spatial extent of deep-water coral reef habitats. Coral reef ecosystems are by nature patchy and fragmented [16], and biodiversity is generally greatest on hard “reef” substrata and lower on soft-bottom inter-reef areas [11], [14]. On shallow-water reefs, high-spatial-resolution multi-spectral images have been used to identify biological and geomorphic features at scales relevant to scientists and marine managers [24]. Identifying similar characteristic features of deep-water coral reefs using remotely-sensed data such as multibeam echosoundings would provide better estimates of the areal extent of coral habitat at regional to global scales and allow for more effective design and implementation of MPAs.

A key consideration in the design of MPAs is “connectivity” between reefs, or the exchange or individuals between reefs via the dispersal of planktonic larvae [16], [25]. However, it is likely that many coral reef connectivity models are missing substantial amounts of reef habitat, reducing the reliability of connectivity models. For example, none of the myriad models of coral reef connectivity on the Great Barrier Reef (GBR) [26]–[29] take account of deep reefs as a possible sources or sinks of coral larvae. If indeed MCEs are linked ecologically to shallow water reefs, data deficiency regarding their location, extent and ecology represents a significant knowledge gap in understanding connectivity between reefs and, by extension, the effectiveness of management strategies to protect the coral reefs from both natural and anthropogenic threats.

The GBR Marine Park is one of the world’s largest MPAs, covering an area of ∼345 000 km^2^. Coral reef habitat is currently regarded as occupying only ∼7% of this area, however this estimate takes little account of submerged reefs (reefs that do not approach the sea surface) that occur on both the shelf-edge [30], [31], [32] and inside the GBR lagoon [32], [33]. Several submerged reefs in the GBR Marine Park have recently been examined using AUV, ROV and SCUBA, and shown them to contain diverse coral reef communities [11], [14]. These observations suggest that total amount of coral habitat within the GBRMP may be substantially underestimated.

Predictive habitat modelling has been used in a variety of ecological applications, including predictive modelling of rare or endangered species [34], [35], conservation planning [36], [37], and predicting climate change impacts [38], [39]. In recent years, there has been significant improvement in the performance of models that require only georeferenced presence-only data [34], [40]. Because direct observations of MCEs are sparse and absence data are generally rare or unreliable, presence-only modelling techniques are well suited to modelling the distribution of mesophotic coral communities. Presence-only techniques have been effectively utilised to predict the distributions of both individual coral species [41] and coral communities [23], [42], [43] in the deep sea, a habitat which contains many parallels to mesophotic coral ecosystems (e.g. inaccessibility, sparse occurrence data). The program Maxent uses [40] maximum entropy techniques to create maps of relative habitat suitability across a geographical area, and has been shown to perform favourably relative to other presence-only modelling techniques, particularly with small sample sizes [44]. Here, we use Maxent to create predictive models of the location and spatial extent of two mesophotic coral reef communities (phototroph-dominated and heterotroph-dominated) in the GBR Marine Park using Maxent. We identify areas where MCE habitat is most likely to occur, and compare the effects of different combinations of geophysical and environmental data layers on model predictions to provide estimates of the location and spatial extent of deep-water coral reef communities within the GBRWHA.

## Methods

This research was conducted under a permit issued by the Great Barrier Reef Marine Park Authority, Townsville, Australia.

### Study Area

**Figure 1:**
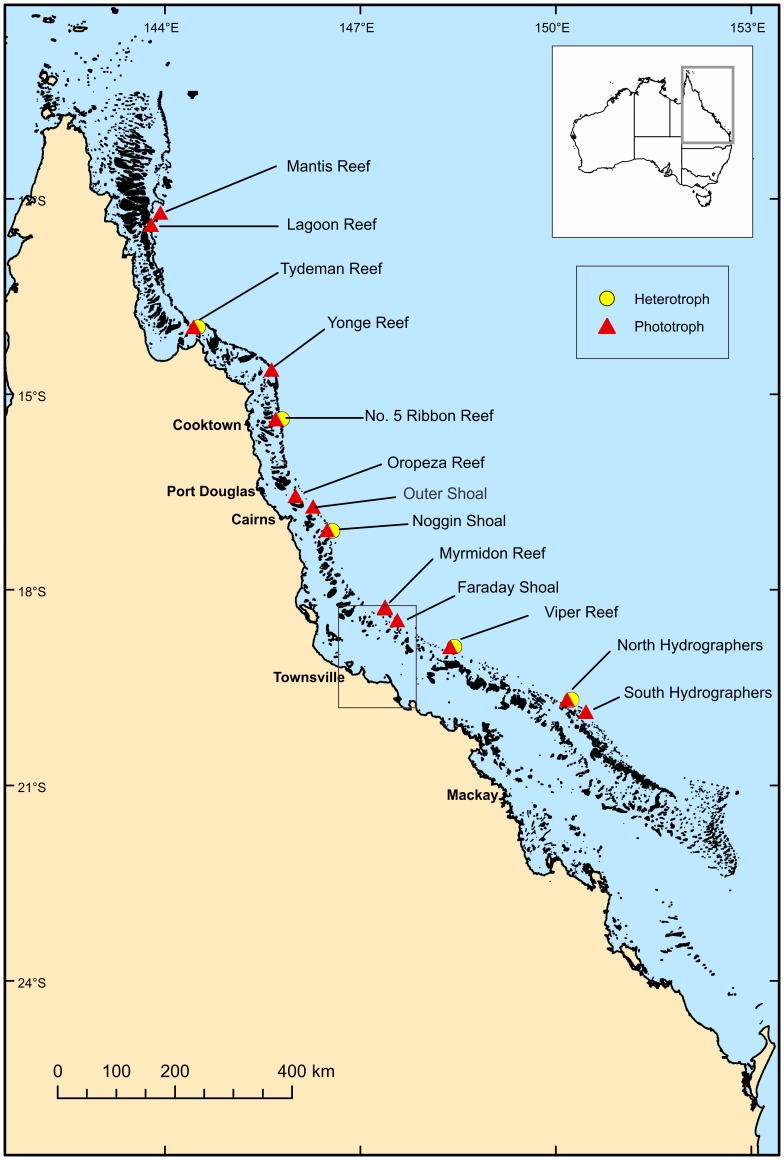
Map of north-east Australia showing location of occurrence records along the Great Barrier Reef. Yellow circles show the location of heterotroph communities and red triangles indicate phototroph communities.

The GBR is composed of over 2900 individual reefs and stretches between approximately latitude 9°S and 25°S (Figure 1). The morphology of the GBR shelf-edge changes from north to south, being generally steeper in the north, and significantly affecting the morphology of the reefs which occur along it [30], [45]. In the northern GBR, long, linear reefs located right on the shelf-edge form a true “barrier reef” system, and narrow submerged reefs occur on their seaward side [30], [31]. The shelf-edge in this region is very steep, and the 500 m isobath is reached only a few hundred metres from the emergent reefs. Below ∼70 m the shelf becomes an almost vertical wall, leaving little space for the development of submerged reefs. South of about 16°06’S, the shelf widens and most reefs are set back from the shelf-edge. This has allowed the development of an extensive series of submerged reefs, which run parallel to the shelf-edge for over 800 km in the central GBR [14], [30], [45], [46]. Submerged reefs also occur inside the GBR lagoon, and these reefs are most abundant in the far north (10–12°S) and also in the south-central GBR (20–23°S), which is consistent with the patterns observed for emergent, shallow-water reefs [32].

### Occurrence Records

**Figure 2:**
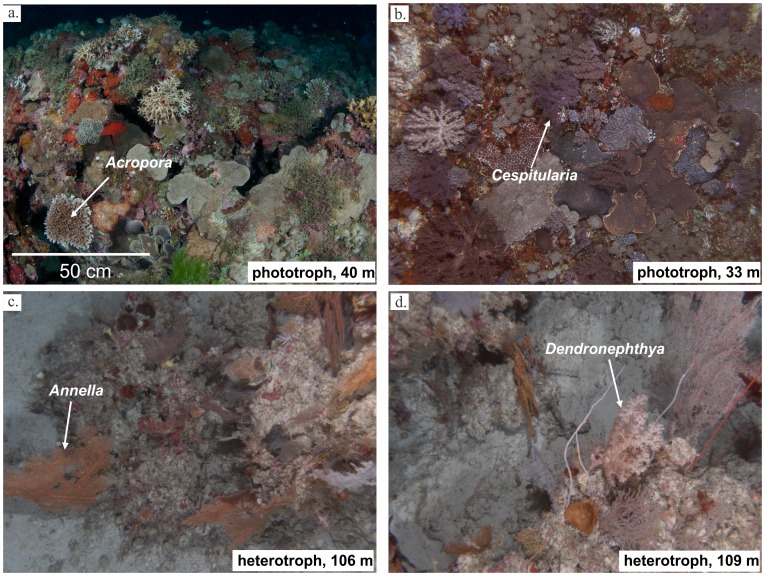
Examples of phototrophic and heterotrophic mesophotic communities on the Great Barrier Reef. Phototrophic communities shown in (a), (b), and heterotrophy communities in (c), (d). Photo (a) by Ed Robert at Mantis Reef, (b), (c) and (d) taken by *Sirius* autonomous underwater vehicle (Australian Centre for Field Robotics) at Hydrographers Passage.

Occurrences of phototroph and heterotroph-dominated MCE communities were derived from georeferenced AUV, ROV and SCUBA surveys conducted from between September 2007 and December 2011 (Figure 1; Table S1). At the species and genus level, MCE community composition on the GBR varies considerably among sites, however there is much greater uniformity among trophic groups of sessile benthic megafauna (SBM) known to occupy particular habitats [11]. To date, sampling of MCEs has been too sparse to identify distribution patterns of individual species, therefore models investigating the extent of MCEs at a GBR-wide scale were conducted using characteristic trophic groups rather than specific species or genera. Models were generated for phototroph-dominated and heterotroph-dominated communities, based on their SBM (Figure 2). Phototroph communities were comprised primarily of taxa which contain symbiotic dinoflagellates (*Symbiodinium* spp.), known as zooxanthellae (Figure 2 a, b). Taxa regularly observed in photosynthetic communities included zooxanthellate Scleractinia (hard corals, including *Porites*, *Acropora*, *Montipora*) and Octocorallia (soft corals, e.g. *Cespitularia*), and phototrophic sponges (e.g. *Carteriospongia*). Heterotrophic communities were dominated by zooxanthellae-free SBM which do not obtain any energy from photosynthate, and included zooxanthellae-free Octocorallia (gorgonians or sea fans, e.g. *Annella*, *Ellisella*), black corals (*Antipathes*) and wire corals (*Cirrhipathes* spp.), as well as a few deep-specialist phototrophs such as *Leptoseris*.

### Environmental Data

Environmental data sets were classified into two main categories: *geophysical* and *environmental*. Geophysical data were all derived from a new high-resolution (100×100 m grid cell) digital elevation model for the GBR called “gbr100” [47]. The five geophysical data layers were used were *depth*, *slope*, *aspect*, *rugosity*, and *geomorphic zones*. *Slope*, *aspect*, *rugosity* and *geomorphic zones* were all derived from the *depth* layer and created in ArcGIS 9.3. *Aspect* and *slope* layers were both created using the relevant tools in the Spatial Analyst toolbox. *Rugosity* was generated using the Focal Statistics tool, which calculates a statistic (standard deviation) on a raster over a specified neighbourhood (in this case 3×3 cells). The *Geomorphic zones* layer was generated using the Benthic Terrain Modeler (BTM) plug-in in ArcGIS, which delineates benthic zone boundaries of the physical landscape [48]. BTM uses an input depth grid to generate Bathymetric Position Index (BPI) datasets through a neighbourhood analysis function. Positive cell values within a BPI dataset denote features that are higher than the surrounding area, such as ridges and pinnacles. Negative cell values within a BPI dataset denote zones that are lower than the surrounding area, such as canyons and gullies. BPI values near zero are either flat areas where the slope is near zero, or areas of constant slope where the slope is significantly greater than zero [48]. Both broad-scale (5×5 pixels) and fine-scale (3×3) BPI grids were generated to calculate geomorphic zones. For this study, grids were reclassified into four basic zones: crests, depressions, flats and slopes, using a 3° slope angle to differentiate between a flat and sloping seafloor.

Environmental data were derived from Bio-ORACLE, a global environmental dataset designed for marine species distribution modelling [48]. Environmental variables selected as potentially important influenced on the distribution of coral reef communities were minimum and mean monthly Chlorophyll A concentration, mean monthly cloud cover, interpolated nitrate concentration, maximum and mean monthly Photosynthetically Active Radiation (PAR), interpolated pH, interpolated Phosphate concentration, and mean, minimum, maximum, and range of monthly sea surface temperature (SST) (see [49] for further information on source of each variable). We also derived one additional environmental variable from the available Bio-ORACLE layers, *SST range*, which was defined as the *SST Maximum* minus *SST Minimum*. For this analysis, it was important to use the finest-scale spatial resolution possible (in this case 100×100 m) in order to resolve potential unmapped reef habitat. Therefore, environmental layers from Bio-ORACLE, available at the scale of 10×10 km, were transformed to match the geophysical data sets (100×100 m) using ArcGIS in order to conform to Maxent’s input data requirements.

### Modelling

Modelling was conducted using Maxent 3.2.19 (http://www.cs.princeton.edu/~schapire/maxent/). Maxent uses the values of environmental or geophysical variables at known species occurrence localities to impose constraints on unknown localities such that the mean of each variable is close to the empirical average at sites where a species is known to occur [50]. We used Maxent for this study because (1) it is accurate with small numbers of occurrence records [40], [50], [51] and (2) reliable absence data are not available for MCEs. Default model parameters used were a convergence threshold of 10^−5^ and a maximum iteration value of 500, which have been shown to achieve good performance on comparable data sets [50]. Model predictions are presented as cumulative probabilities, where the value of a given grid cell is the sum of that cell and all other cells with equal or lower probability [40]. These values can be interpreted as an estimate of the probability of presence under a similar level of sampling effort as that used to obtain the known occurrence data [50]. Duplicate records (where multiple records were present within a single grid cell) were removed from the analysis.

In each model, 70% of the occurrence localities were used as training data, with the remaining 30% used to test model results. The performance of both training and test data sets and of each environmental variable was evaluated using receiver operated characteristic (ROC) curves, with the area under the ROC curve (AUC) reflecting the overall performance of the model and the relative importance of each explanatory environmental variable. In some cases AUC is sensitive to the total spatial extent of the model [52], [53], therefore test gain was also used as a measure of model performance. Gain can be interpreted as the average log probability of the presence samples used to test the model. The total area of MCE habitat in the GBRWHA was estimated using cumulative probability model outputs that had been reclassified into Boolean maps in ArcGIS using two separate thresholds: the 10 percentile training value within Maxent and the lowest presence threshold [44]. The 10^th^ percentile assumes that 10% of occurrence records are erroneous to due factors such as low-resolution environmental data, and therefore excludes all probability values below the highest 10% of records. The lowest presence threshold (LPT) identifies pixels with probability values equal or greater than the value of the lowest occurrence locality, and is therefore a conservative estimate [44]. Model results were also qualitatively tested by comparing model results to empirical observations in areas where extensive sampling effort had occurred, particularly at Hydrographers Passage (see [14]).

Models were run for both phototroph and heterotroph communities using four combinations of environmental data: Geophysical layers only (GEO); Environmental layers only (ENV); all geophysical and environmental layers (GEO-ENV) and the best combination of geophysical and environmental layers as determined by AUC values (BEST). Although Maxent is relatively robust to covariation among environmental variables [40], the BEST layer was chosen to examine if removing covarying layers improved model accuracy. Values chosen as the best combination of variables for phototroph communities were *Depth*, *Mean Chlorophyll*, *SST range*, *Rugosity* and *Geomorphic Zones*. For heterotrophs, the best combination was *Depth*, *rugosity*, *SST range*, and *pH*.

## Results

### Model Evaluation

AUC values for all models were high (>0.96 in all cases), however gain values were substantially higher for models which contained geophysical data (Table S2). Estimates of the total extent of suitable habitat varied substantially depending on input data and independent of the threshold used to define suitable habitat, with particularly large discrepancies observed for heterotrophs (Table 1). Models generated using both geophysical and environmental data tended to overfit predictions of suitable habitat towards regions containing more occurrence records. This pattern was observed in all three models which used environmental data (ENV, GEO-ENV and BEST), and was particularly apparent for heterotrophs. The location of occurrence records did not appear to affect the performance of GEO models.

**Table 1:** Estimated habitat area for phototroph and heterotroph communities using both Lowest Presence (LPT) and 10^th^ Percentile thresholds in square km (km^2^).

	Phototroph - LPT	Phototroph - 10th Percentile	Heterotroph - LPT	Heterotroph - 10th Percentile
**GEO only**	1583	2002	16276	2528
**ENV only**	611	111	18	190
**Both**	1423	414	322	89
**Total GEO**	3006	2416	16598	2617
**Total ENV**	2034	525	340	279

GEO indicates the total area estimated.

**Figure 3:**
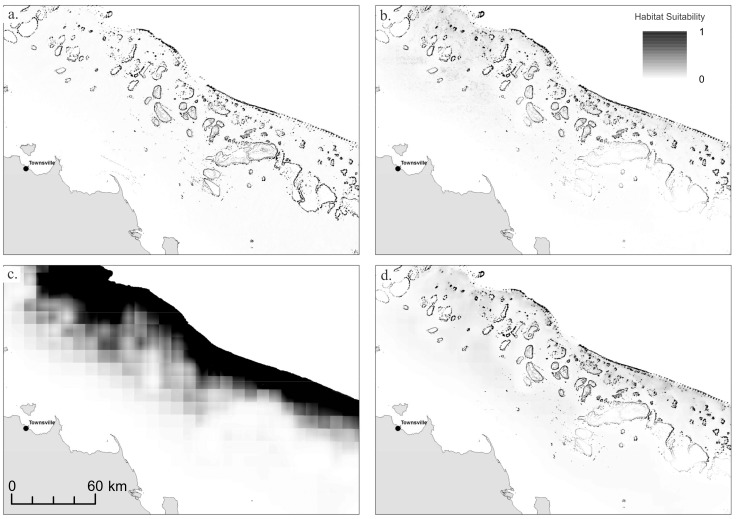
Habitat suitability models for phototrophs for a section of central Great Barrier Reef. (a) GEO; (b) GEO-ENV; (c) ENV; and (d) BEST.

For both phototrophs and heterotrophs, models without any geophysical data were not able to resolve reefs and were therefore generally poor predictors of mesophotic reef habitat, likely because of the comparatively low resolution of ENV-only layers (10×10 km) relative to the scale of reef habitat identified using geophysical layers (Figure 3). Gain was significantly lower in ENV models for both phototrophs and heterotrophs, although this was not reflected in AUC values. However, given that the GBR spans over 13° of latitude, we used models incorporating both geophysical and environmental data to identify whether broad-scale environmental variability could improve predictions of mesophotic coral communities along the entire length of the GBR. Due to the overriding importance of geophysical variables, the results of both GEO-ENV and BEST were very similar, therefore estimates of total habitat area (Table 1) are provided GEO and GEO-ENV models only.

### Phototroph Communities

**Figure 4:**
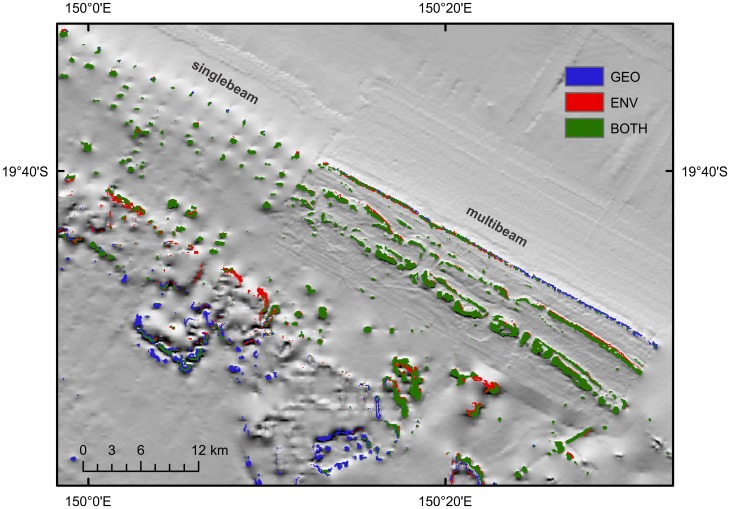
Predictions of phototroph communities in the Hydrographers Passage region, central Great Barrier Reef. GEO only and GEO-ENV both predicted suitable habitat along the outer-self, although model results were more accurate in areas with multibeam (right hand side) compared to singlebeam echosoundings (left). GEO also predicted higher habitat suitability on the deeper flanks of emergent reefs inside the GBR lagoon.

Models of phototroph communities were reasonably consistent regardless of input variables, aside from ENV. Models consistently predicted the occurrence of phototroph MCE communities on the submerged reefs occurring along the outer-shelf and also on the deeper flanks of emergent reefs (Figure 3). However, model predictions were sensitive to the quality of input geophysical data, which varies substantially throughout the GBR (Figure 4). Some sections of the GBR outer-shelf have been mapped with multibeam swath sonar providing 100% coverage of the seafloor at high-resolution, and in these locations the gbr100 grid is of sufficient quality to readily identify the full extent of shelf-edge reefs. However, between these well-mapped sites, much of the shelf-edge has only been surveyed using widely-spaced singlebeam echosounder transects. In regions where singlebeam bathymetry data records a topographic rise due to the presence of a shelf-edge reef, the models show up as patches of phototrophic habitat (Figure 4). Between these transects, the gbr100 grid is relatively smooth due the lack of source bathymetry data and consequently the models do predict suitable habitat at these locations, despite the high probability of shelf-edge reefs being present.

The GEO model predicted greater habitat area than the models using environmental variables. When the LPT was used to determine suitable habitat, the GEO model predicted ∼50% more total habitat area than GEO-ENV (3006 versus 2034 km^2^), with 1423 km^2^ of habitat area predicted by both models (Table 1). Both models consistently predicted suitably habitat occurring on the submerged reefs along the outer-shelf. The largest discrepancy between models occurred on mid-shelf reefs of the central and southern GBR, where GEO predicted the occurrence of phototroph communities on the deeper flanks of emergent mid-shelf reefs as well as on the outer-shelf. In contrast, GEO-ENV suggested that phototroph communities in this region were likely to be restricted to the outer-shelf. When applying 10 percentile threshold, the total amount of habitat area predicted by GEO was similar to LPT (3006 V 2416 km^2^). However, there was a significant difference in the amount of habitat area predicted by models using environmental data (2034 V 525 km^2^). Habitat area estimates using this threshold generally did not predict suitable habitat along the shelf-edge of the central and southern GBR outside of areas where multibeam sonar data are available, and also did not predict mesophotic reef habitat on the deeper flanks of emergent reefs.

Geophysical layers *Geomorphic zone, Slope* and *Rugosity* were the most explanatory variables for phototrophs (Table S2). The most predictive environmental variable was *SST range*, although no environmental variables were very predictive for phototrophs.

### Heterotroph Communities

**Figure 5:**
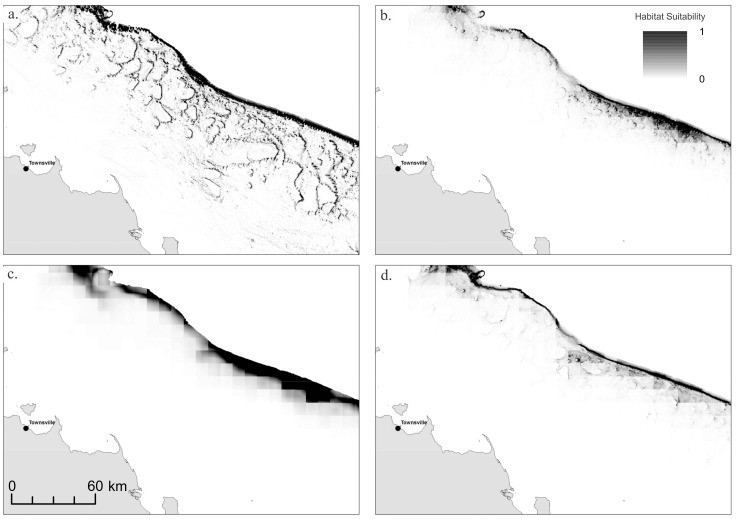
Habitat suitability models for heterotrophs for a section of central Great Barrier Reef. (a) GEO; (b) GEO-ENV; (c) ENV; and (d) BEST.

**Figure 6:**
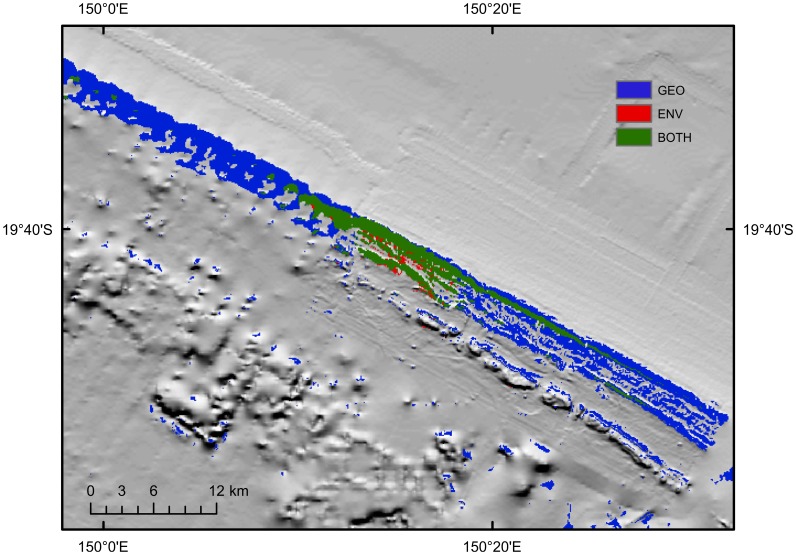
Predictions of heterotroph communities for the same region of Hydrographers Passage, central Great Barrier Reef. GEO models consistently predicted suitable habitat on the outer-shelf, and low suitability in shallower waters. Models using environmental data overfitted the predictions towards the location of occurrence records.

Heterotroph communities showed greater variability in both the location and spatial extent of habitat suitability among modelling techniques. As with phototrophs, the ENV model performed poorly, and the inclusion of environmental data (in addition to geophysical data) appeared to reduce the accuracy of models compared to the GEO-model (Figure 5). The GEO model predicted high habitat suitability in the deeper waters along the outer-shelf, and also on the deeper flanks of emergent reefs (Figure 5a). Despite high AUC values indicating good model performance, models incorporating environmental data (Figure 5 b, c, d) consistently indicated low habitat suitability in regions with few occurrence records. This effect was particularly pronounced in the region around Hydrographers Passage, which contained the greatest number of occurrence records (Figure 6). Estimates of the total spatial extent of heterotroph habitat varied widely depending upon input variables and thresholds from over 16 000 km^2^ (GEO LPT) to less than 300 km^2^ (ENV 10^th^ percentile), although GEO consistently predicted greater heterotroph habitat than models using environmental variables (Table 1).

## Discussion

These results indicate that coral reefs may be far more extensive and exist across a broader range of habitats than previously realised, and provides important new information for assessing the vulnerability of coral reef ecosystems to global climate change. This study represents the first attempt to quantify the spatial extent of deep reef habitat anywhere in the world, although [54] previously used depth (30–100 m depth range) to identify areas that may *potentially* support MCEs in United States territorial waters. Their study indicated that MCEs may occur over large areas, both on continental and insular shelves, and may occupy a greater areal extent than shallow-water coral reefs. Similarly, Harris et al. [32] recently used the gbr100 bathymetry model to conduct a geomorphologic analysis of submerged banks on the GBR, and concluded that these features occupy over 41 000 km^2^ of the GBRWHA, 160% of the area of emergent, shallow water reefs. Although not all these banks would support mesophotic coral communities, these results confirm earlier geological studies indicating that submerged reefs are common features of continental shelves and around oceanic islands in many of the world’s coral reef provinces. Although many of these earlier studies focused on the geomorphology of submerged reefs, they point to the potentially significant proportion of coral reef habitat which has received very little attention from ecologists or marine managers.

Our results indicate that coral reef communities are likely to occur on submerged reefs and on the deeper flanks of emergent reefs both along the GBR outer-shelf and inside the lagoon. Given the unique biodiversity already reported from MCEs in the GBR [11], [14] and their potential importance as refugia for coral reef species, these habitats should receive greater research interest from both scientists and managers. Our results indicate that high-resolution geophysical data is well suited to identifying MCE communities, and is effective even without other environmental data such as sea temperature. However, unlike many terrestrial studies, the patchy nature of coral reefs means that geophysical data of sufficient resolution to delineate reefs are critically important. Given that direct in-situ observations of submerged reefs are not feasible given time and funding constraints, modelling efforts such as those presented here will provide important tools for marine managers, allowing greater consideration MCEs in management decisions and MPA design. Furthermore, although this study focuses primarily on submerged coral reefs, it is likely these results would be transferable to other marine ecosystems. For example, kelp forests replace coral reefs as the dominant habitat-forming benthos in southern Australia; however, despite significant research effort on shallow-water kelp reefs, deeper kelp forest reefs currently represent a significant knowledge gap [55].

Many reefs on the GBR are relatively small, often 1–10 km diameter. In the northern GBR shelf-edge reefs are also very narrow, with many submerged reefs only tens of metres wide. This presents difficulties in resolving reefs, and therefore for the ability of the models to detect MCE habitat. Widely-spaced single-beam echosoundings were generally not sufficient to resolve reefs, and resulted in underestimation of total habitat area. More accurate estimates of total extent of MCE habitat would be best achieved via collection of multibeam bathymetry for areas of the reef where only single-beam soundings are currently available, and would be of significant value for regional-scale marine habitat modelling. It is also important to note that submerged reefs may well support a higher percentage of coral cover, on average, than emergent reefs. Predictive habitat modelling conducted at Hydrographers Passage using 5×5 m grid cell bathymetry [32] suggested that mesophotic coral communities in that region occupy ∼55% of the area of submerged banks. Many emergent reefs feature extensive sandy lagoons, not conducive to high coral cover ([56] estimate mean coral cover on emergent reefs in the GBR at ∼29%). Currently, the GBRWHA is regarded as supporting ∼20 000 km^2^ of reef habitat, of which ∼30% is likely to be covered by live corals [57]. If ∼50% of submerged banks support living corals, as these studies suggest, then the GBR actually supports significantly more coral cover than currently appreciated. Further investment in the collection systematic, high-resolution multibeam data would enable more accurate predictions of the exact location and spatial extent of deep reef habitat throughout the entire GBRWHA.

In this study, models run using environmental variables were consistently overfitted to the input data. Although Maxent has consistently performed favourably relative to other presence-only modelling techniques such as GARP [44], it can sometimes bias predictions towards areas with more input occurrence records, particularly at higher probability thresholds [58]. In this study, models incorporating environmental data consistently failed to predict distributions in regions with fewer occurrence records regardless of the environmental variables used, while models using geophysical data only seemed more robust to the spatial distribution of occurrence records. Although other modelling techniques such as GARP are less prone to overfitting, they have the drawback of generalising distribution predictions, and are therefore not suitable for delineating reefs. Occurrence records used in this study are widely distributed along the GBR but were still relatively sparse owing to the lack of observations on MCEs. Obtaining a greater spatial distribution of occurrence records may help alleviate the problem of overfitting of model predictions when using environmental variables.

Another issue encountered during this study was selecting suitable environmental variables. This problem was exacerbated by the spatial scale of environmental layers (10×10 km) compared to geophysical layers (100×100 m). Furthermore, some environmental correlations indicated to be important by Maxent are likely to be casual in the field. For example, AUC values suggested that mean chlorophyll should be an important factor controlling the distribution of heterotrophs. Heterotrophic octocorals feed on phytoplankton, and previous studies have shown that their taxonomic richness is greatest in areas of highest productivity [59]. However, paradoxically, habitat predicted to be highly suitable for heterotrophs in the present analysis was correlated with low chlorophyll values. Although shallow-water chlorophyll concentration is lowest in the clear, oceanic waters of the outer-shelf, it does not necessarily reflect plankton availability to deep MCE communities. Seafloor chlorophyll, therefore, may be a more effective predictor of suitable heterotroph habitat. Although the GEO models appear to be relatively good at predicting suitable habitat for broad trophic groups, the availability of more detailed environmental data would likely improve model performance, and allow more detailed modelling at higher taxonomic resolutions. Such modelling would also require more mesophotic faunal occurrence records to be collected before it could materially improve our ability to predict the location of and structure of phototrophic and heterotrophic MCE communities.

These results suggest that coral reef habitat within the GBRWHA is likely to be more extensive than current estimates. Moreover, given that submerged reefs have been reported from continental shelves and oceanic islands in many locations around the world [60], it is likely that many coral reef provinces support extensive mesophotic coral reefs that are currently undocumented. The models presented here are clearly transferable to other parts of the world provided that sufficient quality bathymetry data are available, and could be used to generate testable hypotheses about where MCEs occur as the basis for planning for field sampling. So verified, model predictions could then be used in the planning for networks of MPAs, particularly those aiming to identify areas less likely to be exposed to threats associated with global climate change. Given that MCEs may be buffered from many of the threats shallow coral reefs currently face, identifying and preemptively protecting mesophotic coral reefs from threats such as over-fishing should be an urgent priority for marine resource managers. Although direct observation of MCEs is difficult, our results show that increased focus on collecting broad-scale geophysical data, particularly high-resolution multibeam bathymetry, and small, well-focused field campaigns to verify faunal predictions, will provide sufficient detail to identify submerged reefs and associated coral reef ecosystems which can then be incorporated into MPAs. The use of robust models such as these thus means that a precautionary approach to MPA design in the absence of complete information could be far more comprehensive and cost-effective that it would be without it.

## Supporting Information

Table S1 **Location and survey method of each occurrence record used in this study.** Locations are shown in Figure 1.
(DOCX)

Table S2 **AUC Values and jackknife measure of variable importance for GEO, ENV, and GEO-ENV models for both phototrophs and heterotrophs.**
(DOCX)
