# Supplementary material for: Predicting the Location and Spatial Extent of Submerged Coral Reef Habitat in the Great Barrier Reef World Heritage Area, Australia
Source: PLoS One. 2012 Oct 30;7(10):e48203. doi: 10.1371/journal.pone.0048203 (PMC3484119; doi:10.1371/journal.pone.0048203)
Supplement: Table S1 — Location and survey method of each occurrence record used in this study. Locations are shown in Figure 1. (DOCX) [file pone.0048203.s001.docx]

Table S1: Location and survey method of each occurrence record used in this study. Locations are shown in Figure 1.

| Type | North | East | Longitude | Latitude | Location | AUV | SCUBA | ROV |
| --- | --- | --- | --- | --- | --- | --- | --- | --- |
| Phototroph | 166083.3 | 8648371 | 143.9315 | -12.20965 | Mantis Reef |  | X | X |
| Phototroph | 150548.2 | 8627645 | 143.7867 | -12.395156 | Lagoon Reef |  | X | X |
| Heterotroph | 230817 | 8454426 | 144.5084 | -13.967759 | Tydeman Reef |  | X | X |
| Phototroph | 230813.5 | 8454369 | 144.5084 | -13.968271 | Tydeman Reef |  | X | X |
| Phototroph | 353192.2 | 8383633 | 145.637 | -14.616652 | Yonge Reef |  | X | X |
| Heterotroph | 370848.1 | 8299727 | 145.7966 | -15.375996 | No. 5 Ribbon Reef |  |  | X |
| Phototroph | 370789.7 | 8299573 | 145.7961 | -15.377386 | No. 5 Ribbon Reef | X |  |  |
| Phototroph | 370815.1 | 8299543 | 145.7963 | -15.377652 | No. 5 Ribbon Reef | X |  |  |
| Phototroph | 393288 | 8169124 | 145.9998 | -16.557554 | Oropesa Reef |  | X |  |
| Phototroph | 449252 | 8121032 | 146.5253 | -16.99088 | Outer Shoal |  | X |  |
| Phototroph | 453458 | 8108801 | 146.5625 | -17.10502 | Noggin Shoal |  |  | X |
| Heterotroph | 454311.3 | 8110284 | 146.5706 | -17.091398 | Noggin Pass | X |  |  |
| Phototroph | 453830.8 | 8109989 | 146.566 | -17.094056 | Noggin Pass | X |  |  |
| Phototroph | 453892.4 | 8109977 | 146.5666 | -17.094158 | Noggin Pass | X |  |  |
| Phototroph | 540027.1 | 7981519 | 147.3787 | -18.255372 | Myrmidon Reef | X |  |  |
| Phototroph | 539926.3 | 7981431 | 147.3777 | -18.256171 | Myrmidon Reef | X |  |  |
| Phototroph | 539665.8 | 7978832 | 147.3753 | -18.279663 | Myrmidon Reef | X |  |  |
| Phototroph | 559629 | 7958834 | 147.5647 | -18.459944 | Myrmidon Reef | X |  |  |
| Heterotroph | 651975.1 | 7912732 | 148.4427 | -18.871858 | Faraday Shoal |  | X |  |
| Heterotroph | 651923.4 | 7912612 | 148.4422 | -18.87295 | Viper Reef | X |  |  |
| Phototroph | 651460 | 7912024 | 148.4379 | -18.878292 | Viper Reef | X |  |  |
| Phototroph | 651545.2 | 7911929 | 148.4387 | -18.879148 | Viper Reef | X |  |  |
| Heterotroph | 840477.6 | 7822093 | 150.2468 | -19.667231 | North Hydrographers Passage | X |  |  |
| Heterotroph | 840404.6 | 7822007 | 150.2461 | -19.668022 | North Hydrographers Passage | X |  |  |
| Heterotroph | 840304 | 7821886 | 150.2451 | -19.66913 | North Hydrographers Passage | X |  |  |
| Heterotroph | 840232.4 | 7821797 | 150.2445 | -19.669943 | North Hydrographers Passage | X |  |  |
| Heterotroph | 840147.8 | 7821687 | 150.2437 | -19.670951 | North Hydrographers Passage | X |  |  |
| Heterotroph | 840083.7 | 7821592 | 150.2431 | -19.671818 | North Hydrographers Passage | X |  |  |
| Heterotroph | 840009.1 | 7821486 | 150.2424 | -19.672788 | North Hydrographers Passage | X |  |  |
| Heterotroph | 839952.8 | 7821384 | 150.2419 | -19.673719 | North Hydrographers Passage | X |  |  |
| Heterotroph | 839935.2 | 7821353 | 150.2417 | -19.674004 | North Hydrographers Passage | X |  |  |
| Heterotroph | 839847 | 7821200 | 150.2409 | -19.675397 | North Hydrographers Passage | X |  |  |
| Heterotroph | 839644.2 | 7820819 | 150.2391 | -19.678867 | North Hydrographers Passage | X |  |  |
| Heterotroph | 839582.3 | 7820701 | 150.2385 | -19.679947 | North Hydrographers Passage | X |  |  |
| Heterotroph | 839543.9 | 7820625 | 150.2381 | -19.680635 | North Hydrographers Passage | X |  |  |
| Heterotroph | 839474.2 | 7820491 | 150.2375 | -19.681858 | North Hydrographers Passage | X |  |  |
| Heterotroph | 839417.6 | 7820384 | 150.237 | -19.682837 | North Hydrographers Passage | X |  |  |
| Heterotroph | 839172.9 | 7819891 | 150.2347 | -19.68732 | North Hydrographers Passage | X |  |  |
| Heterotroph | 839137.6 | 7819820 | 150.2344 | -19.687969 | North Hydrographers Passage | X |  |  |
| Heterotroph | 839072.6 | 7819699 | 150.2338 | -19.689076 | North Hydrographers Passage | X |  |  |
| Heterotroph | 839024.1 | 7819589 | 150.2334 | -19.690071 | North Hydrographers Passage | X |  |  |
| Heterotroph | 839020.9 | 7819514 | 150.2334 | -19.690749 | North Hydrographers Passage | X |  |  |
| Heterotroph | 839018.2 | 7819425 | 150.2333 | -19.69155 | North Hydrographers Passage | X |  |  |
| Heterotroph | 840873.7 | 7819021 | 150.2511 | -19.694877 | North Hydrographers Passage | X |  |  |
| Heterotroph | 840759.1 | 7818851 | 150.25 | -19.696434 | North Hydrographers Passage | X |  |  |
| Phototroph | 840409.8 | 7818517 | 150.2468 | -19.699506 | North Hydrographers Passage | X |  |  |
| Phototroph | 840407.9 | 7818450 | 150.2468 | -19.700113 | North Hydrographers Passage | X |  |  |
| Phototroph | 840381.7 | 7818432 | 150.2465 | -19.700281 | North Hydrographers Passage | X |  |  |
| Phototroph | 862618.9 | 7799487 | 150.462 | -19.86721 | South Hydrographers Passage | X |  |  |
| Phototroph | 862620.6 | 7799478 | 150.462 | -19.867285 | South Hydrographers Passage | X |  |  |
| Phototroph | 862526.7 | 7799406 | 150.4612 | -19.867955 | South Hydrographers Passage | X |  |  |
| Phototroph | 862463.7 | 7799383 | 150.4606 | -19.868178 | South Hydrographers Passage | X |  |  |
| Phototroph | 862614.5 | 7799374 | 150.462 | -19.86823 | South Hydrographers Passage | X |  |  |
| Phototroph | 862502.7 | 7799346 | 150.4609 | -19.868499 | South Hydrographers Passage | X |  |  |
