# Supplementary material for: Predicting the Location and Spatial Extent of Submerged Coral Reef Habitat in the Great Barrier Reef World Heritage Area, Australia
Source: PLoS One. 2012 Oct 30;7(10):e48203. doi: 10.1371/journal.pone.0048203 (PMC3484119; doi:10.1371/journal.pone.0048203)
Supplement: Table S2 — AUC Values and jackknife measure of variable importance for GEO, ENV, and GEO-ENV models for both phototrophs and heterotrophs. (DOCX) [file pone.0048203.s002.docx]

Table S2: AUC Values and jackknife measure of variable importance for GEO, ENV, and GEO-ENV models for both phototrophs and heterotrophs.

|  | **Phototroph GEO** | **Phototroph ENV** | **Phototroph GEO-ENV** | **Heterotroph GEO** | **Heterotroph ENV** | **Heterotroph GEO-ENV** |
| --- | --- | --- | --- | --- | --- | --- |
|  |  |  |  |  |  |  |
| **Training AUC** | 0.9617 | 0.9737 | 0.9984 | 0.9926 | 0.997 | 0.9986 |
| **Test AUC** | 0.9964 | 0.9565 | 0.9974 | 0.9898 | 0.9962 | 0.9974 |
| **Test Gain** | 4.44 | 1.98 | 5.32 | 3.97 | 1.98 | 4.74 |
| **10th Percentile Logistic Threshold** | 0.3616 | 0.23 | 0.5455 | 0.3426 | 0.08 | 0.453 |
| **Lowest Presence Threshold** | 0.3 | 0.16 | 0.15 | 0.05 | 0.052 | 0.11 |
|  |  |  |  |  |  |  |
| **AUC for each variable** |  |  |  |  |  |  |
| Depth | 0.7381 |  | 0.738 | 0.9468 |  | 0.9377 |
| Slope | 0.9516 |  | 0.9523 | 0.9072 |  | 0.8387 |
| Aspect | 0.6638 |  | 0.5507 | 0.8474 |  | 0.8443 |
| Rugosity | 0.952 |  | 0.9524 | 0.9055 |  | 0.8832 |
| Zones | 0.9804 |  | 0.9507 | 0.7153 |  | 0.6884 |
| Chlorophyll Mean |  | 0.8386 | 0.8384 |  | 0.9804 | 0.9805 |
| Chlorophyll Min |  | 0.7806 | 0.7807 |  | 0.9784 | 0.9784 |
| Cloud Mean |  | 0.7899 | 0.7898 |  | 0.979 | 0.9787 |
| Nitrate Mean |  | 0.6839 | 0.6837 |  | 0.9555 | 0.9555 |
| PAR Max |  | 0.6714 | 0.6712 |  | 0.9192 | 0.9192 |
| PAR Mean |  | 0.5848 | 0.5917 |  | 0.9733 | 0.9736 |
| pH |  | 0.5544 | 0.5542 |  | 0.9381 | 0.9372 |
| Phosphate Mean |  | 0.7057 | 0.7056 |  | 0.9432 | 0.9432 |
| SST Max |  | 0.696 | 0.6971 |  | 0.9099 | 0.9094 |
| SST Mean |  | 0.7851 | 0.7852 |  | 0.9403 | 0.9403 |
| SST Min |  | 0.7941 | 0.7941 |  | 0.9427 | 0.9429 |
| SST Variance |  | 0.8779 | 0.8784 |  | 0.9695 | 0.9695 |
|  |  |  |  |  |  |  |
| **Jackknife of variable importance** |  |  |  |  |  |  |
| Aspect | 0.0051 |  | 0.0394 | 0.9007 |  | 0.73 |
| Depth | 0.3799 |  | 0.3785 | 1.7852 |  | 2.7587 |
| Rugosity | 2.7621 |  | 2.7889 | 1.4336 |  | 2.6934 |
| Slope | 2.7776 |  | 2.9149 | 1.3922 |  | 2.4164 |
| Zones | 3.141 |  | 2.1763 | 0.6609 |  | 1.5757 |
| Chlorophyll Mean |  | 0.5317 | 0.5322 |  | 2.7588 | 2.1291 |
| Chlorophyll Min |  | 0.3611 | 0.3625 |  | 2.6926 | 1.381 |
| Cloud Mean |  | 0.5375 | 0.5371 |  | 2.4178 | 0.6463 |
| Nitrate Mean |  | 0.2419 | 0.2416 |  | 2.129 | 2.24 |
| PAR Max |  | 0.184 | 0.1838 |  | 1.3803 | 2.028 |
| PAR Mean |  | -0.1093 | -0.0981 |  | 0.586 | 0.995 |
| pH |  | -0.2228 | -0.2231 |  | 2.2399 | 0.7505 |
| Phosphate Mean |  | 0.2348 | 0.2349 |  | 2.0273 | 1.3939 |
| SST Max |  | 0.3353 | 0.3361 |  | 1.3948 | 1.8422 |
| SST Mean |  | 0.6068 | 0.6072 |  | 1.843 | 1.7851 |
| SST Min |  | 0.5119 | 0.5116 |  | 1.7826 | 2.105 |
| SST Variance |  | 0.964 | 0.9644 |  | 2.1018 | 0.4036 |
